# Supplementary material for: Longitudinal Association between Uric Acid and Incident Sarcopenia
Source: Nutrients. 2023 Jul 11;15(14):3097. doi: 10.3390/nu15143097 (PMC10384494; doi:10.3390/nu15143097)
Supplement: Supplementary file 1 [file nutrients-15-03097-s001.zip › nutrients-2467656-supplementary.pdf]

Table S1. Baseline characteristics of included and excluded participants in this study.

	Total	Included	Excluded
N	17708	5086	12622
Uric acid, mg/dl	4.46 ± 1.26	4.43 ± 1.23	4.48 ± 1.29
Demographic			
Age, years	59.05 ± 10.15	58.36 ± 8.34	59.33 ± 10.78
Residence			
Urban	4564 (25.83)	842 (16.61)	3772 (29.54)
Rural	13105 (74.17)	4227 (83.39)	8878 (70.46)
Education level			
Primary school or below	11755 (66.38)	3534 (69.48)	8221 (66.38)
Middle school	3652 (20.62)	1059 (20.82)	3652 (20.62)
High school or above	2301 (12.99)	493 (9.69)	1808 (14.32)
Marital status			
Married and living with spouse	14170 (80.17)	4407 (86.65)	9763 (77.55)
Others	3505 (19.83)	679 (13.35)	2826 (22.45)
Health behavior			
Drinking			
Yes	5767 (32.87)	1663 (32.74)	4104 (32.93)
No	11776 (67.13)	3417 (67.26)	8359 (67.07)
Smoking			
Yes	4862 (28.76)	1516 (29.88)	3346 (28.27)
No	12045 (71.24)	3557 (70.12)	8488 (71.73)
Sleep duration, h	6.37 ± 1.89	6.37 ± 1.82	6.37 ± 1.93
Chronic diseases			
Diabetes Mellitus	2115 (12.04)	750 (14.75)	1365 (10.94)
Hypertension	9369 (53.34)	2049 (40.29)	7320 (58.66)
Depression	5239 (29.59)	1623 (31.91)	3616 (28.65)
Dyslipidemia	3646 (20.59)	1406 (27.64)	2240 (17.75)
Pulmonary disease	1781 (10.18)	493 (9.74)	1288 (10.36)
Kidney disease	1106 (6.34)	349 (6.91)	757 (6.10)
Cardiovascular disease	2093 (11.98)	586 (11.59)	1507 (12.14)
Arthritis	5773 (32.94)	1788 (35.24)	3985 (32.01)
Biochemical parameters			
Creatinine, mg/dl	0.78 ± 0.23	0.77 ± 0.18	0.78 ± 0.27
eGFR, ml/(min×1.73 m2)	108.22 ± 29.76	108.37 ± 28.73	108.11 ± 30.54
HDL, mg/dl	50.84 ± 15.32	50.64 ± 15.07	50.99 ± 15.52
LDL, mg/dl	115.99 ± 34.91	117.19 ± 34.80	115.06 ± 34.96
CRP, mg/L	2.80 ± 7.63	2.43 ± 6.60	3.08 ± 8.32

Continuous variables are presented as the mean ± standard deviation, and categorical variables are expressed as numbers (percentages).

Abbreviations: eGFR, estimated glomerular filtration rate; HDL-C, high-density lipoprotein cholesterol; LDL-C, low-density lipoprotein cholesterol; CRP, high sensitivity C-reactive protein; SMI, skeletal muscle mass index.

Table S2. Hazard ratios and 95% confidence intervals for incident sarcopenia according to the sex-specific quartiles of uric acid and uric acid as a continuous variable after excluding participants with hyperuricemia. (n=4819)

Outcome	Cases	Incidence rate, per 1000 person-years	Model 4	P-value
Males				
Continuous variable (UA per 1 mg/dl)	349	42.66	0.82 (0.73-0.93)	0.002
UA Quartiles				
Q1 (≤4.06mg/dl)	127	60.13	1	
Q2 (4.06-4.81mg/dl)	96	44.24	0.80 (0.61-1.06)	0.124
Q3 (4.81-5.67mg/dl)	84	38.18	0.73 (0.54-0.98)	0.037
Q4 (≥5.67mg/dl)	42	24.74	0.52 (0.35-0.75)	0.001
Females				
Continuous variable (UA per 1 mg/dl)	171	16.82	0.92 (0.75-1.12)	0.44
UA Quartiles				
Q1 (≤3.29mg/dl)	46	17.26	1	
Q2 (3.29-3.87mg/dl)	49	18.46	1.01 (0.66-1.55)	0.936
Q3 (3.87-4.57mg/dl)	44	16.48	0.98 (0.62-1.54)	0.955
Q4 (≥4.57mg/dl)	32	14.68	0.92 (0.56-1.53)	0.761

Data are presented as HRs (95%CIs).

Model 4: adjusted age, residence, educational level, marital status, drinking status, smoking status, nighttime sleep duration, DM, hypertension, depression, dyslipidemia, pulmonary disease, kidney disease, cardiovascular disease, arthritis, eGFR, CRP, HDL-C and LDL-C.

Abbreviations: HRs, hazards ratios; CIs, confidence intervals; DM, diabetes mellitus; eGFR, estimated glomerular filtration rate; HDL-C, high-density lipoprotein cholesterol; LDL-C, low-density lipoprotein cholesterol; CRP, high sensitivity C-reactive protein.

Table S3. Hazard ratios and 95% confidence intervals for incident sarcopenia according to the sex-specific quartiles of uric acid and uric acid as a continuous variable after excluding participants with eGFR≤ 60 ml/min per 1.73 m2. (n=5007)

Outcome	Cases	Incidence rate, per 1000 person-years	Model 4	P-value
Males				
Continuous variable (UA per 1 mg/dl)	364	42.68	0.90 (0.81-0.99)	0.047
UA Quartiles				
Q1 (≤4.06mg/dl)	126	59.82	1	
Q2 (4.06-4.81mg/dl)	95	43.98	0.79 (0.60-1.05)	0.111
Q3 (4.81-5.67mg/dl)	81	37.39	0.71 (0.53-0.96)	0.028
Q4 (≥5.67mg/dl)	62	29.59	0.62 (0.44-0.87)	0.006
Females				
Continuous variable (UA per 1 mg/dl)	182	17.29	1.05 (0.89-1.24)	0.553
UA Quartiles				
Q1 (≤3.29mg/dl)	46	17.29	1	
Q2 (3.29-3.87mg/dl)	49	18.49	1.01 (0.66-1.54)	0.946
Q3 (3.87-4.57mg/dl)	44	16.53	0.98 (0.62-1.54)	0.945
Q4 (≥4.57mg/dl)	43	16.86	1.08 (0.67-1.74)	0.741

Data are presented as HRs (95%CIs).

Model 4: adjusted age, residence, educational level, marital status, drinking status, smoking status, nighttime sleep duration, DM, hypertension, depression, dyslipidemia, pulmonary disease, kidney disease, cardiovascular disease, arthritis, eGFR, CRP, HDL-C and LDL-C.

Abbreviations: HRs, hazards ratios; CIs, confidence intervals; DM, diabetes mellitus; eGFR, estimated glomerular filtration rate; HDL-C, high-density lipoprotein cholesterol; LDL-C, low-density lipoprotein cholesterol; CRP, high sensitivity C-reactive protein.
